# Natural persistence of the coastal plant *Glehnia littoralis* along temperate sandy coasts

**DOI:** 10.1038/srep42784

**Published:** 2017-02-17

**Authors:** Hong-Xiao Yang, Jian-Min Chu, Xiao-Shan Liu

**Affiliations:** 1 Qingdao Engineering Research Center for Rural Environment, College of Resources and Environment, Qingdao Agricultural University, Chengyang, Qingdao, Shandong Province 266109, China; 2 Key Laboratory of Tree Breeding and Cultivation, State Forestry Administration, Research Institute of Forestry, Chinese Academy of Forestry, Beijing 100091, China

## Abstract

We studied germination behaviors and persistence mechanism of wild *Glehnia littoralis*, a typical coastal species at temperate sandy coasts of the North Pacific Ocean, and tested the hypothesis that the coastal plants may have evolved special seeds adapting to the coasts, by which they recruit and persist easily, occupying the coasts as ideal habitats. In the Shandong Peninsula, China, we investigated temperature and moisture conditions of coast sand in relation to germination and evaluated effects of sand burial, seawater immersion and sowing time on germination. When germination began, daily dawn temperatures of sand were about 10 °C and daily noon temperatures were about 25 °C; the temperatures were not different in the sand <8 cm deep. The sand at these depths showed a significant difference in moisture contents. The seeds exhibited large germination rates if sand burial was at depths >= 3 cm and winter freezing was kept longer than 2.5 months. Seeds experiencing seawater immersion were able to germinate well. These evidences suggest that *G. littoralis* has evolved special seeds adapting to seawater dispersal and specific season rhythm. By the seeds, *G. littoralis* occupies temperate sandy coasts as ideal habitats to persist.

The worldwide coastal vegetation suffers from increasing habitat loss, tourist trampling and artificial dams^1^,^2^,^3^. Many coastal species making up the vegetation are declining, and local or global extinction is ongoing^4^,^5^. They are irreplaceable stabilizers or maintainers of coastal ecosystems, and also rare resources to provide humans with herbal medicine, fibre and other necessities^6^,^7^,^8^. *Glehnia littoralis* that can be used as herbal medicine to treat lung diseases of humans is a typical species growing at temperate sandy coasts around the North Pacific Ocean^9^,^10^,^11^,^12^. Unfortunately, it is now in danger of extinction for habitat loss and exploitation^13^,^14^,^15^. Human efforts are needed to save the species from extinction. Such coastal specialists may have established special relationships with pertaining coasts^16^,^17^. Knowledge of these relationships is useful for making a plan of species conservation^18^,^19^.

Wild *G. littoralis* is distributed from Eastern China, the Korea peninsula to Western Canada and America^7^,^16^,^20^. It is not a dominant species of coastal vegetation, and the distribution is narrowly limited to supratidal zones of fore-dunes in widths of ca. 10 or 20 m^16^,^21^. Adults of the species are often not taller than 40 cm, with annual leaves and fruiting branches, and with perennial roots and short stems. The roots can grow deeper than 40 or 50 cm to survive fierce winter. Several decades ago, the species was widespread at fore-dunes of sandy coasts, but at present, is very rare and endangered, only appearing at a few remote coasts free of tourism and exploitation^15^,^21^. The Chinese government has enrolled it as a species deserving strict conservation, and because of significant effects in curing lung diseases, it has been transplanted inland for field cultivation^12^,^22^,^23^,^24^,^25^. Nevertheless, we doubt that the *ex situ* cultivation can provide the species with ideal conditions for persistence.

Supratidal zones of temperate sandy coasts are characterized by environmental factors such as rich sand matrix, long winter and, in particular, occasional inundation during seawater surges^26^,^27^,^28^. As a survivor with long history, *G. littoralis* may have adapted to these factors and evolved a special life^7^,^17^. The life of a plant species always involves seeds and seedlings^29^,^30^,^31^. *G. littoralis* seeds were found to be buoyant^13^,^17^,^32^,^33^. However, persistence of wild *G. littoralis* was unknown. Given this background, we designed field surveys and experiments to recognize germination behaviors and persistence mechanism of wild *G. littoralis*. Furthermore, we tested the hypothesis that the typical coastal species has evolved special seeds to adapt to temperate coasts, by which it recruits and persists easily, occupying temperate sandy coasts as ideal habitats.

## Results

### Temperature and moisture conditions for germination

**Figure 1:**
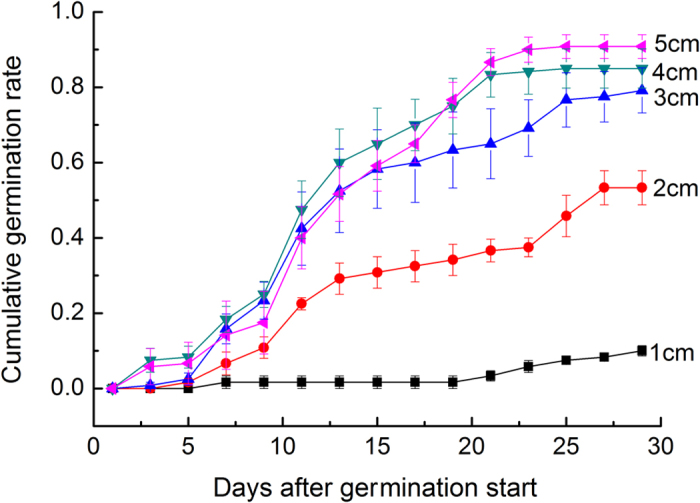
Cumulative germination rates of *G. littoralis* seeds buried at different depths (1–5 cm).

When the germination began, daily dawn and noon temperatures of the sand were ca. 10 °C and 25 °C, respectively. The germination lasted for about a month (Fig. 1). During the process, the daily dawn and noon temperatures were 12.9 (mean) ± 2.1 (standard deviation) °C and 26.9 ± 8.6 °C, respectively. The maximum daily dawn temperature was 16 °C, and the maximum daily noon temperature was 40 °C. Two-way ANOVA indicated that these temperatures were not different among the four layers, but varied significantly with time changing (Table 1).

The results of two-way ANOVA indicated that moisture contents varied significantly with time, and nearly significantly among the four layers (Table 1). The moisture contents were 0.055 (mean) ± 0.076 (standard deviation) at the 0–2 cm layer, with a variation coefficient of 1.374 (standard deviation/mean); 0.071 ± 0.071 at the 2–4 cm layer, with a variation coefficient of 1.000; 0.084 ± 0.072 at the 4–6 cm layer, with a variation coefficient of 0.848; and 0.076 ± 0.046 at the 6–8 cm layer, with a variation coefficient of 0.608. The top layer was the driest and most unsteady, perhaps because of intense and variable evaporation. The 6–8 cm layer was not the moistest perhaps because rains during the early spring were often too weak to infiltrate so deep.

### Effects of burial depths, seawater immersion and sowing time on germination

The cumulative germination rates responded differently to the burial depths (Table 1). The germination rates were significantly low at the depths <= 2 cm (Fig. 1), but were fairly high at the depths >= 3 cm. These results demonstrate that the depths favorable for germination were not less than 3 cm, perhaps because moisture contents were high and steady at these depths.

**Figure 2:**
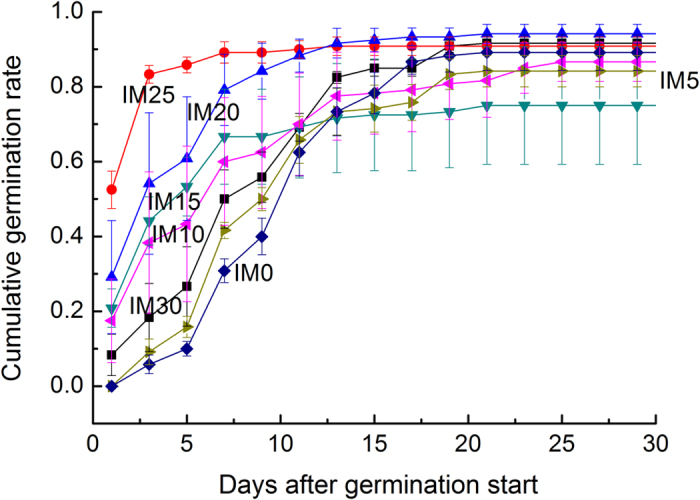
Cumulative germination rates of *G. littoralis* seeds experiencing pre-germination seawater immersion for some days. IM0, not immersed by seawater; IM5, immersed for 5 days; IM10, immersed for 10 days; IM15, immersed for 15 days; IM20, immersed for 20 days; IM25, immersed for 25 days; IM30, immersed for 30 days.

Seawater immersion might have no effects on final cumulative germination rates (Table 1), which could exceed 60%, but dynamics of the germination differed obviously among these immersion levels (Fig. 2). These results indicate that seawater immersion might not promote the maximum germination rates of *G. littoralis* seeds but could activate the seeds for a swift germination. The pre-germination immersion for 25 days was the best in urging the germination.

**Figure 3:**
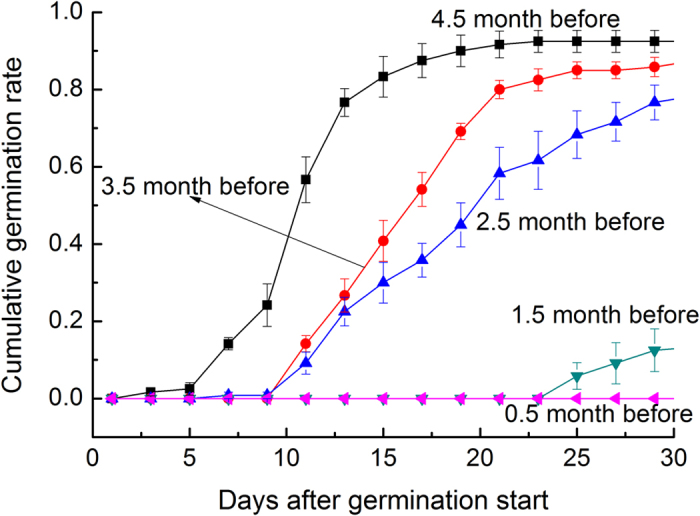
Cumulative germination rates of *G. littoralis* seeds sown some months before spring germination. 0.5 month before: 0.5 month before the germination start; 1.5 month before: 1.5 month before the germination start; 2.5 month before: 2.5 month before the germination start; 3.5 month before: 3.5 month before the germination start; 4.5 month before: 4.5 month before the germination start.

Sowing time dominated the cumulative germination rates (Table 1). The earlier the seeds were sown in winter, the higher the final cumulative germination rates (Fig. 3). Outdoor freezing for longer than 2.5 months was necessary to arouse *G. littoralis* seeds for a swift and successful germination. The finding suggests that *G. littoralis* seeds have the property of winter-dormancy, and most of them remain dormant until winter provides them a freezing longer than 2.5 months.

## Discussion

Seeds of wild *G. littoralis* require appropriate temperatures and moisture for germination. After daily dawn and noon temperatures of sand rise to ca. 10 and 25 °C, *G. littoralis* seeds get ready for germination. We observed that if sown at the depths >= 3 cm, they germinate easily. The four sand layers showed a significant difference in moisture contents, other than in temperatures. Moisture contents were the lowest and most unstable at the 0–2 cm depth, which was adverse to germination. At the depth >= 3 cm, moisture contents became high and steady adequately, feasible for seed germination.

It was reported on that seeds of *G. littoralis* have the property of winter-dormancy^13^,^34^. This study provides details to further understand this. In wilderness, seeds of wild *G. littoralis* can hardly germinate unless aroused by fierce freezing longer than 2.5 months. This property is a protective adaptation of this species to the long winter of the temperate zone. Adults of the species bear seeds in late summer, when temperatures are favorable. Nevertheless, the seeds refuse to germinate that time. Because of winter-dormancy, they choose to germinate after winter, thus new seedlings emerge in early spring, gaining more time to grow strong to survive the coming winter.

*Glehnia littoralis* seeds can keep vigorous or turn more vigorous after seawater immersion, unlike most terrestrial plants^35^,^36^,^37^. This is a critical adaptation to seawater dispersal^33^,^38^,^39^. Supratidal zones are characterised by occasional seawater inundation^8^,^40^. Around the North Pacific Ocean, extreme storms often take place around winter to cause violent seawater surges^41^,^42^. As a result, seeds of *G. littoralis* at supratidal zones can be inundated and dispersed^17^,^43^,^44^. They keep afloat for many days, like seeds of *Eryngium maritimum, Cakile maritime* and *Canavalia rosea*^17^,^38^,^39^. They are immersed in seawater, whereas await a chance to land at supratidal zones again^17^. After they land at favorable environment, they may germinate and grow, continuing life cycles of the species. In case *G. littoralis* seeds were disabled during the immersion, this species would have difficulties in recruiting, and not persist for long along supratidal zones.

These germination behaviors confirm the hypothesis that the typical coastal specialist *G. littoralis* have evolved special seeds to adapt to temperate sandy coasts, at least including seawater dispersal, season rhythm and sandy matrix. By coupling the germination with the environment, we conceived the mechanism of wild *G. littoralis* for dispersal and persistence along temperate sandy coasts.

Adults of wild *G. littoralis* give birth to mature seeds before autumn. Although temperatures remain favorable that time, inherent dormancy sets back immediate germination of them. Storms can take place around winter in temperate seas, unlike in tropical ones^42^,^45^. They inevitably cause seawater surging, which may co-occur and overlap with high astronomical tides to make extreme surges. The surges can inundate supratidal zones for several hours. On this occasion, *G. littoralis* seeds are immersed in seawater and begin dispersing with seawater current^17^,^46^. Because of high buoyancy, they keep afloat during the immersion, and are able to return to supratidal zones with the same surge or another surge some days later^17^,^47^. With seawater, they disperse in short and long distances, and may settle at supratidal zones again. After settling at supratidal zones of temperate sandy coasts, they get the opportunity to began new lives. They are buried by moving sand with winds. During the burial, fierce winter provides them with effective freezing. After winter, they get aroused and ready for germination. In early spring, the daily dawn temperature rises to ca. 10 °C, and some seeds receive the signal to germinate^48^. Those on or in surface sand (< 3 cm deep) can hardly germinate, perhaps because of unfavorable moisture conditions. From spring to autumn, winds are relatively mild and temperatures are favorable, thus the seedlings grow as fast as possible. Several months later, taproots of them grow deep and strong enough to tolerate the first winter they will encounter. After the winter, they revive to grow. By perennial roots and winter-resistant buds, they repeat the revival and growth year after year, bearing seeds for species recruitment and persistence^49^.

The temperate sandy coasts around the North Pacific Ocean provide wild *G. littoralis* with ideal conditions, such as sand burial and freezing in windy winter, surging seawater for seed dispersal and activation, and calm growing season from spring to autumn. For such a provision, wild *G. littoralis* occupies temperate sandy coasts around the north Pacific Ocean as original habitats. After transplanted to inland, the species cannot disperse normally as along coasts, and has to lead a spoiled life, which may be adverse to species persistence^50^,^51^. Since *G. littoralis* is a typical coastal specialist depending on seawater dispersal, it ought to be conserved in temperate sandy coasts, where seawater dispersal is convenient, season rhythm is suitable and sand matrix is available. Additionally, with the warming of global climates, this species may shift its distribution area northward because long cold winter is necessary for it to germinate and regenerate, and it may be driven extinct from some warm temperate regions such as the Shandong Peninsula^52^.

## Methods

### Field surveys

Field surveys were conducted in 2012 at sandy coasts of the Shandong Peninsula (N 35.85–36.70°, E 120.05–121.20°, near Qingdao), where *G. littoralis* still exists along supratidal zone (Table 2). Seeds of the species germinated in early April. During the germination, we measured field temperatures of the sand with glass thermometers one time every 10 days, and the measurement lasted for a month until the germination almost ceased, i.e., four times in total. Daily dawn temperatures were measured just before sunrise, and daily noon temperatures were measured at 14:00. Each time of the measurement was done at three locations randomly chosen from an area of ca. 200 m^2^ where seedlings emerged in clumps, and at each location, four sand layers were investigated, covering the depths of 0–2, 2–4, 4–6 and 6–8 cm.

We sampled sand four times from these layers to measure moisture contents, one time every 10 days from the germination start. Every time, we randomly chose 10 points in the ca. 200 m^2^ area to sample sand. At these points, we collected sand vertically with a drill and divided the sand into the four layers. Sand from the same layer from the 10 points was together lumped and sealed in a waterproof plastic bag as a composed sample. Within 3 h, we weighed the samples and dried them in an electronic oven (70 °C for 2 days). We weighed the dried samples and calculated their gravimetric moisture contents, dividing the moisture mass (mass of the wet sample minus that of the dried sample) by the mass of the dried sample.

### Experiments

In August 2012, we collected mature seeds of the species (carrying seed capsule) from undisturbed sandy coasts and air-dried them for storage. In November, we began the experiment one at a coast (E 120.93°, N 36.41°) to identify sand depths favorable for germination (Table 2). We loaded 20 wood boxes measuring 40 cm (length) × 20 cm (width) × 20 cm (height) with drainage holes at the bases with sand from the coast. These boxes were divided into five equal groups, one group containing four boxes. We sowed the seeds in these boxes, and sowing depths were one of the levels: 1, 2, 3, 4 and 5 cm. One box group (4 boxes) corresponded to one level of the sowing depths. In each box, 30 seeds were evenly sown at the equal depth. After sowing, we gently watered the sand within the boxes with collected rainfall. Then, we left the boxes outdoor and spread a mesh 2 m above to prevent birds (other than snow, rain and light). Monitoring was performed everyday. Since seedlings emerged, we counted new ones every 2 days, which we timely marked with fine toothpicks. The counting and recording lasted for a month until germination almost ceased.

In August 2013, we collected seeds again and air-dried them, to prepare the experiments two and three (Table 2). From October 17, we began to immerse seeds in seawater, one batch every 5 days. By November 16, we prepared seven batches of seeds ever immersed for 30, 25, 20, 15, 10, 5 and 0 days. During the immersing, we replaced the seawater every 5 days. On November 16, we fished out and sowed the seeds in the same boxes as the mentioned above, and placed them outdoor at the coast. Each immersion level included four boxes (replicates), where 30 seeds were evenly sown at the depth of 3 cm (we found it feasible for germination).

On November 20, 2013, the experiment three started to examine the effects of sowing time on germination (Table 2). We sowed the newly collected seeds but not subjected to any seawater immersion within the same boxes every month until March 20, 2014. The experiment included five levels of sowing time: November 20, December 20, January 20, February 20 and March 20. Every level included four boxes (as replicates), and in each box, 30 seeds were evenly sown at the depth of 3 cm. After sowing, we watered and monitored the boxes as in the experiment one. On April 6, 2014, germination began. Then, new seedlings were counted and marked every 2 days for a month. The authors comply with Convention on the Trade in Endangered Species of Wild Fauna and Flora.

### Data analyses

Using two-way ANOVA, we examined the differences in daily dawn temperatures, daily noon temperatures and moisture contents among the four sand layers and the changing time points. The sand layers were four depth levels: 0–2, 2–4, 4–6 and 6–8 cm. The changing time points included four levels in the germination process: start of the germination, 1/3 of the germination period (the 10th day), 2/3 of the germination period (the 20th day) and the end of germination. We calculated cumulative germination rates over the germination process, which were seedling number until a certain day divided by the number of initially sown seeds. Using one-way ANOVA, we checked effects of sowing depth, seawater immersion and sowing time on the final cumulative germination rates. Data analyses were completed using Origin 8.0 (OriginLab, Northampton, USA) and R 3.2 (R Core Team 2015).

## Additional Information

**How to cite this article:** Yang, H.-X. *et al*. Natural persistence of the coastal plant *Glehnia littoralis* along temperate sandy coasts. *Sci. Rep.* **7**, 42784; doi: 10.1038/srep42784 (2017).

**Publisher's note:** Springer Nature remains neutral with regard to jurisdictional claims in published maps and institutional affiliations.

## Figures and Tables

**Table 1:** Results of statistical analyses.

Item	Factor	Levels	Replicates	Statistical method	*F* value	Significance (*p*)
Daily dawn temperature	Sand layer	4	3	Two-way ANOVA	*F*_3,41_ = 0.654	0.585
	Time changing	4	3		*F*_3,41_ = 115.326	<0.001***
Daily noon temperature	Sand layer	4	3	Two-way ANOVA	*F*_3,41_ = 0.794	0.505
	Time changing	4	3		*F*_3,41_ = 4895.635	<0.001***
Moisture content	Sand layer	4	1	Two-way ANOVA	*F*_3,9_ = 2.927	0.092*
	Time changing	4	1		*F*_3,9_ = 85.945	*p* < 0.001***
Final cumulative germination rate	Burial depth	5	4	One-way ANOVA	*F*_4,15_ = 65.03	<0.001***
	Seawater immersion	7	4	One-way ANOVA	*F*_6,21_ = 0.865	0.536
	Sowing time	5	4	One-way ANOVA	*F*_4,15_ = 153	<0.001***

Note: *p < 0.1; ** *p* < 0.01; ***p < 0.001.

**Table 2:** Brief description of the methods.

Methods	Brief description
Field surveys	During the germination period, we measured daily dawn and noon temperatures of coast sand. Measurements were conducted at four layers (covering the depths of 0–2, 2–4, 4–6 and 6–8 cm), one time every 10 days. At the same time, we sampled sand from these layers to measure gravimetric moisture contents.
Experiment one	This experiment was conducted to identify sand depths favorable for seed germination. We collected seeds in autumn and sowed them in winter in sand at the depths of 1, 2, 3, 4 and 5 cm.
Experiment two	This experiment was designed to assess effects of seawater immersion on seed germination. We prepared seven batches of seeds that experienced pre-germination seawater immersion for 0, 5, 10, 15, 20, 25 and 30 days. In winter, we sowed them in sand at the depth of 3 cm.
Experiment three	The experiment was used to examine effects of sowing time on seed germination. We sowed seeds in sand at the depth of 3 cm; the sowing time was, respectively, November 20, December 20, January 20, February 20 and March 20.
